# Polymorphisms in the *RANK/RANKL* Genes and Their Effect on Bone Specific Prognosis in Breast Cancer Patients

**DOI:** 10.1155/2014/842452

**Published:** 2014-03-05

**Authors:** Alexander Hein, Christian M. Bayer, Michael G. Schrauder, Lothar Häberle, Katharina Heusinger, Reiner Strick, Matthias Ruebner, Michael P. Lux, Stefan P. Renner, Rüdiger Schulz-Wendtland, Arif B. Ekici, Arndt Hartmann, Matthias W. Beckmann, Peter A. Fasching

**Affiliations:** ^1^Department of Gynecology and Obstetrics, University Breast Center Franconia, University Hospital Erlangen, Friedrich-Alexander University Erlangen-Nuremberg, Comprehensive Cancer Center Erlangen-EMN, Erlangen, Germany; ^2^Institute of Diagnostic Radiology, University Hospital Erlangen, Friedrich-Alexander University Erlangen-Nuremberg, Comprehensive Cancer Center Erlangen-EMN, Erlangen, Germany; ^3^Institute of Human Genetics, University Hospital Erlangen, Friedrich-Alexander University Erlangen-Nuremberg, Comprehensive Cancer Center Erlangen-EMN, Erlangen, Germany; ^4^Institute of Pathology, University Hospital Erlangen, Friedrich-Alexander University Erlangen-Nuremberg, Comprehensive Cancer Center Erlangen-EMN, Erlangen, Germany

## Abstract

The receptor activator of NF-*κ*B (RANK) pathway is involved in bone health as well as breast cancer (BC) pathogenesis and progression. Whereas the therapeutic implication of this pathway is established for the treatment of osteoporosis and bone metastases, the application in adjuvant BC is currently investigated. As genetic variants in this pathway have been described to influence bone health, aim of this study was the prognostic relevance of genetic variants in *RANK* and *RANKL*. Single nucleotide polymorphisms in *RANK*(*L*) (rs1054016/rs1805034/rs35211496) were genotyped and analyzed with regard to bone metastasis-free survival (BMFS), disease-free survival, and overall survival for a retrospective cohort of 1251 patients. Cox proportional hazard models were built to examine the prognostic influence in addition to commonly established prognostic factors. The SNP rs1054016 seems to influence BMFS. Patients with two minor alleles had a more favorable prognosis than patients with at least one common allele (HR 0.37 (95% CI: 0.17, 0.84)), whereas other outcome parameters remained unaffected. rs1805034 and rs35211496 had no prognostic relevance. The effect of rs1054016(*RANKL*) adds to the evidence that the RANK pathway plays a role in BC pathogenesis and progression with respect to BMFS, emphasizing the connection between BC and bone health.

## 1. Introduction

Breast cancer treatment becomes more and more individualized. Additionally to directed and undirected therapies, diagnostic tests of the tumor and the host are being integrated into the therapy plan. Recently a meta-analysis of more than 20,000 women who were treated with or without bisphosphonates reports an absolute reduction in breast cancer specific mortality after 10 years of 3.1%. Most of the recurrences that were prevented seemed to be bone related [1]. Besides bisphosphonates modern therapies such as directed antibody therapy against RANKL (receptor activator of NF-*κ*B ligand) with denosumab [2] are in the focus of current research efforts as the RANK (receptor activator of NF-*κ*B) pathway has been described to be involved in both bone metabolism and breast cancer pathogenesis and progression [3–9].

RANKL is a member of the tumor necrosis factor superfamily. It binds to its cytokine receptor RANK and initiates a series of organ specific responses. In the bone it mediates the formation and activity of osteoclasts [10]. In the breast it is thought to induce progesterone mediated cell proliferation [4, 6, 7]. This molecular pathway is of special interest as the monoclonal antibody denosumab as a drug is used not only for the treatment of bone loss but also for the prevention of skeletal related events in breast cancer patients with metastatic disease to the bone [11] and is under investigation for the treatment of breast cancer in the adjuvant setting. In this paper we describe the effects of genetic variants in genes of this pathway on the risk to develop bone metastases.

Bone metastases are a common event in metastatic breast cancer patients. In about 25% to 40% the bone is the first site of the metastatic disease [12]. Spread of the primary tumor is a multifactorial process, which is not completely understood. Concerning common patient and tumor characteristics some predictors have been described. These include high number of involved lymph nodes, larger tumor size, and a positive estrogen receptor status [13–15]. With regard to the molecular characterization luminal tumors seemed to have the highest risk for developing bone metastases [16]. Concerning the host, it has been discussed that bone turnover might be associated with an increased risk for the development of bone metastases [17, 18].

Many studies have identified genetic variants in the genes *RANKL*, *RANK,* and *OPG* (osteoprotegerin) to have an influence on bone mineral density and the risk for bone fractures [19–25]. Therefore it can be assumed that according to a host's germline genotype there are different patterns of bone turnover and bone resorption.

Aim of our study was therefore to investigate the influence of known genetic variations in *RANKL* and *RANK* on the prognosis of breast cancer patients, especially on the bone metastasis-free survival.

## 2. Material and Methods

### 2.1. Patient Cohort

The Bavarian Breast Cancer Cases and Controls Study (BBCC) was designed as a case-control and cohort study to investigate genetic and epidemiological factors as risk and prognostic factors for breast cancer. Cases and controls were collected from 2002 to 2008 [26–29]. The BBCC is part of the Breast Cancer Association Consortium (BCAC) and has taken part in the validation of confirmed breast cancer susceptibility single nucleotide polymorphisms (SNPs) [30–38]. This paper reports the cases within this study only. Briefly, primary breast cancer patients who were treated at the University Breast Center for Franconia were asked to take part in the study. This analysis presented here included cancer patients whose diagnosis was no longer than 1 year before the blood was drawn. The collection of follow-up data for the analysis closed on August 31, 2011. The patients were followed up for cancer recurrence and mortality. Mortality data were obtained from the German death registry. Data on recurrences were documented prospectively for patients attending the Franconia Breast Center for follow-up care, in accordance with the data management standards of the German certification board for breast centers (http://www.onkozert.de/) and the European Society of Mastology (EUSOMA). Patients who were not attending the Breast Center were contacted annually by mail. Patients who did not respond to the letter were contacted by phone. The median follow-up period for the cohort was 7.4 years. This study was approved by the Ethics Committee of the Friedrich-Alexander-University Erlangen-Nuremberg.

### 2.2. Isolation of DNA from Blood

DNA was extracted from 8 mL blood samples using a genomic DNA purification kit (Puregene; Gentra Systems, Minneapolis, Minnesota, USA/Qiagen Ltd., Hilden, Germany) with modifications. Briefly, after initial centrifugation, the white blood cell layer was removed and added to RBC lysis buffer, pH 7.3, containing 0.15 M NH_4_Cl, 0.01 M K_2_CO_3_, and 0.1 M Na-EDTA. After 10 min incubation, the cells were centrifuged at 2,000 g and incubated with 3 mL cell lysis buffer containing 20 mM Tris pH 7.4, 15 mM Na-EDTA, and 1% SDS and treated with RNase A and proteinase K (all from Sigma- Aldrich Chemie Ltd., Schnelldorf, Germany). The proteins were precipitated with 1 mL of protein precipitation solution (Puregene), and the DNA was precipitated by the addition of isopropanol, washed with 70% ethanol, dried, solubilized with a Tris-EDTA buffer, pH 7.5, quantitated using a spectrophotometer, and stored at −80°C. Using this methodology, an average of 70–100 lg of DNA per patient was obtained.

### 2.3. Genetic Marker Selection

We have selected SNPs with potential functional relevance by altering the amino acid sequence (missense variants) or being located in regulatory parts of the gene, one being located in *RANKL* and two within *RANK*. rs1054016 lies in the 3′ untranslated region (UTR) of *RANKL, *rs18054016 (*RANK*) results in an exchange from alanine to valine (C/T; Ala192Val), and rs35211496 (*RANK*) results in an amino acid exchange from histidine to tyrosine (C/T; His141Tyr).

### 2.4. Genotyping

Real-time polymerase chain reaction tests were purchased from Applied Biosystems (ABI, Foster City, California, USA/Applera Deutschland Ltd., Darmstadt, Germany) for the SNPs in accordance with the National Center for Biotechnology Information terminology (NCBI, http://www.ncbi.nlm.nih.gov/). Reactions were prepared with 2x Genotyping MasterMix in accordance with the manufacturer's instructions and performed on a sequence detection system (Prism 7900HT; ABI) using standard thermal cycling conditions.

### 2.5. Statistical Considerations

Bone metastasis-free survival (BMFS) was defined as the time from the date of diagnosis of breast cancer to either the date of diagnosis of bone-metastasis or the date of censoring. Patients who were lost to follow-up within 10 years or died within 10 years were censored at the last day they were known to be bone metastasis-free or the date of death. A patient who was bone metastasis-free 10 years after diagnosis was censored at that date. Disease-free survival (DFS) and overall survival (OS) were defined in a similar way.

Cox proportional hazards (PH) models were used to investigate the prognostic value of the SNPs rs1054016, rs1805034, and rs35211496 on BMFS, DFS and OS, respectively, in addition to well-known prognostic factors. For each SNP, a Cox PH model with the following predictors was set up: age at diagnosis (continuous), body mass index (BMI, continuous), tumor stage (ordinal; pT0, pT1,…, pT4), grading (ordinal; G1, G2, G3), nodal status (categorical; positive, negative), tumor type (categorical; ductal, lobular, other), and the selected SNP (categorical; three genotypes). The estrogen receptor status (ER) was incorporated into the model as stratification factor (ER-positive, ER-negative) because the proportional hazards assumption was violated for ER. Patients with missing outcome variable were excluded from the analysis. Hazard ratios (HRs) for genotypes were estimated. *P* values were not corrected for multiple testing. Missing predictor values were imputed using single “best guesses” (median value of continuous predictors, the most common value of categorical or ordinal categorical predictors) based on nonmissing data across all subjects. Survival rates were estimated for interesting results using the method of Kaplan-Meier.

The proportional hazards assumptions in the SNP models were checked using the Grambsch and Therneau method [39].

All of the tests were two-sided, and a *P* value of <0.05 was regarded as statistically significant. Calculations were carried out using the R system for statistical computing (version 3.0.1; R Development Core Team, Vienna, Austria, 2013).

## 3. Results

A total of 1251 patients were included in the analysis. 34 patients with more than 20% missing values, 44 patients without survival data, and 5 patients with metastases at date of diagnosis had to be excluded. 86.7% of all patients had complete patient and tumor characteristics and 98.4% of all patients had complete SNP information. The percentage of missing values in each variable was below 2% except of BMI (4.2%) and grading (5.1%). The missing values were imputed as described above.

The patient population resembled a heterogeneous cohort of breast cancer patients with most patients having T1 tumors, being nodal negative and having a hormone receptor positive tumor. Patient characteristics are summarized in Table 1.

Genotyping results are shown in Table 2. The two SNPs rs1054016 (RANKL) and rs1805034 (RANK) had a minor allele frequency of 42.2% and 47.8%, respectively, and were relatively common polymorphisms in our population. The minor allele frequency of rs35211496 (*RANK*) was less common with 17.3%. All genotypes were within the Hardy Weinberg equilibrium.

**Figure 1:**
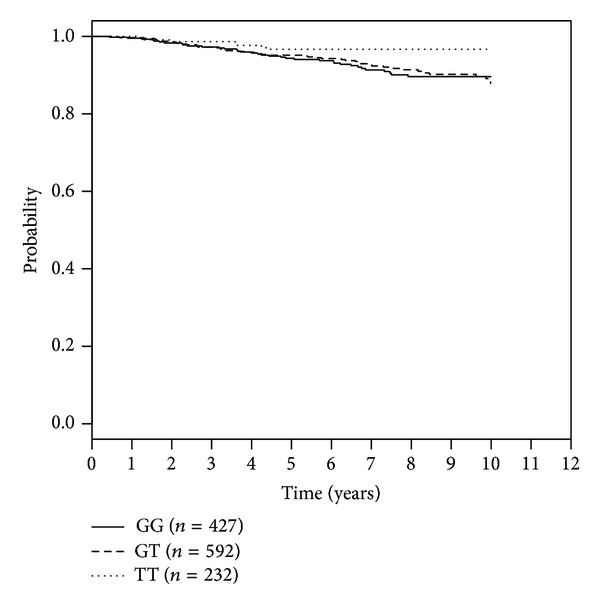
Kaplan-Meier curves for rs1054016 and bone metastasis-free survival according to genotypes.

**Figure 2:**
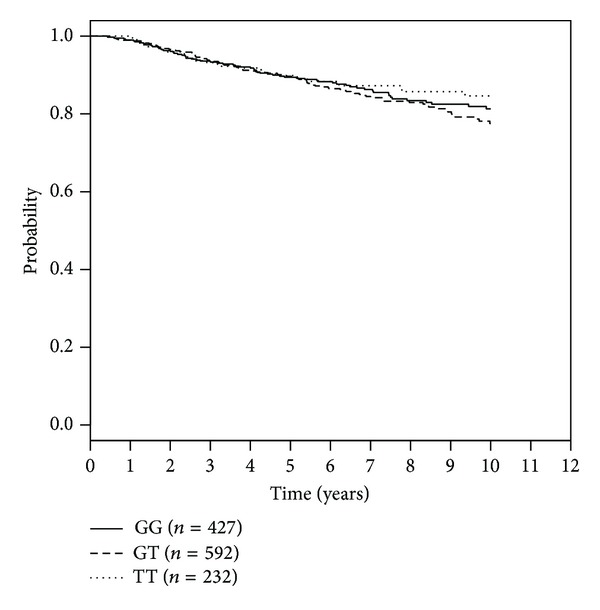
Kaplan-Meier curves for rs1054016 and progression-free survival according to genotypes.

**Figure 3:**
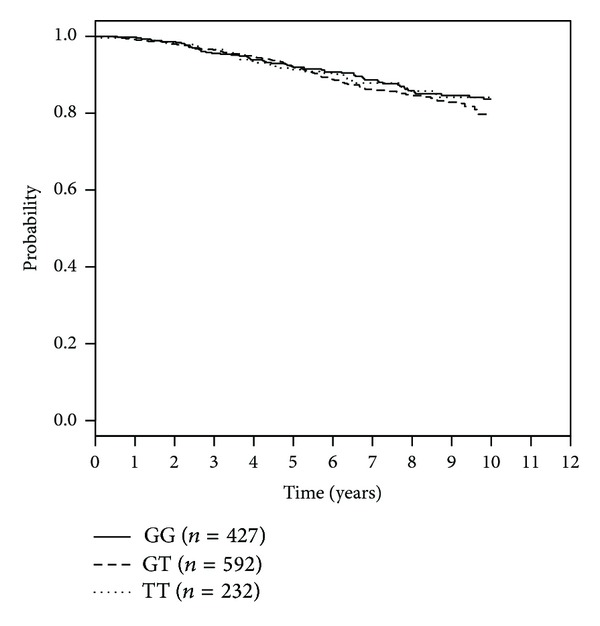
Kaplan-Meier curves for rs1054016 and overall survival according to genotypes.

The SNP rs1054016 seems to influence the time to bone-metastasis. Patients with two minor alleles had a better prognosis than patients with at least one common allele (Table 3). The Kaplan Meier curve for bone metastasis-free survival and rs1054016 is shown in Figure 1. The hazard ratio was 0.37 (95% CI: 0.17 to 0.84) for patients with a TT genotype compared with patients with a GG genotype. No association could be seen with regard to distant disease-free survival or overall survival. The Kaplan-Meier curves for this analysis are shown in Figures 2 and 3.

We could not show any prognostic differences with regard to the other SNPs (Table 3).

## 4. Discussion

The minor allele of the SNP rs1054016 (*RANKL*) seems to reduce the time to bone-metastasis. No association could be made with distant disease-free survival and overall survival. Two other analyzed SNPs in RANKL and RANK did not show any prognostic relevance in our cohort.

With regard to prognosis it has been recently shown that RANK expression of the tumor is associated with disease-free survival and overall survival in the univariate analysis [40]. In this study on neoadjuvantly treated breast cancer patients RANK was scored on a tissue microarray. High expression was seen in tumors with higher grading, low hormone receptor expression, and positive HER2 status. In multivariate analysis RANK did not maintain statistical significance [40]. In a study analyzing data from gene expression chips, similar results were seen with patients having tumors with high mRNA levels of RANK having a less favorable skeletal disease-free survival [41]. Additionally low levels of OPG mRNA showed a worse prognosis as well [41]. Another study with a small sample size reported that lower levels of RANKL expression correlated with poorer overall survival [42].

Our study has examined the effect of genetic variants on bone metastasis-free survival, disease disease-free, and overall survival. Looking at these separate analyses, the effect on prognosis was only seen with regard to bone metastasis-free survival. Concerning the hypothesis that genetic variants of the RANK/RANKL/OPG system might act through an effect of the skeleton, this result underlines the hypothesis of the RANK pathway being associated specifically with osseous metastases [43].

rs1054016 has been described in other studies to have an influence on age at menarche. Individuals being homozygous for the rare allele T had a menarche that was about 4 months earlier than individuals being homozygous for the common allele G [44]. Another study implied some role in longevity [45]. Other studies examining femoral neck compression strength [46], susceptibility to rheumatoid arthritis [47], or breast cancer [48] did not see any association.

As rs1054016 lies in the 3′UTR region of *RANKL*, genetic variants could have an influence on posttranscriptional gene expression, possibly having an influence on mRNA stability. Furthermore, 3′UTR regions are reported to contain miRNA responsive elements [49].

There are some limitations to our study. We have chosen a candidate SNP approach, selecting genes of interest and focusing on genetic variants that might have the greatest impact on the biological processes concerning transcription, translation, and function of the protein. Looking at association studies in breast and ovarian cancer this approach was not very successful and the results that were produced could not often be replicated [50]. Out of more than 75 validated breast cancer risk SNPs, only one was discovered with a candidate gene approach [8, 31, 51]. Genome-wide association studies with a more agnostic approach have identified most of the robust findings with regard to many phenotypes. Many of these genetic variants are located in introns and regions, which do not seem to have an effect on amino acid exchanges or other evident functional role. Slowly it is learnt that many of the variants might lie in regions of epigenetic relevance or at miRNA binding sites. In our study, however, only candidate SNPs with a hypothetical impact on prognosis were selected. With regard to a conclusion that rs1054016 might have an impact only on bone specific prognosis and not on other prognostic outcome measures, it has to be kept in mind that our analysis did not address the issue of competing risks. Other limitations include the retrospective design of our study, including breast cancer patients, who have been treated heterogeneously. However, the data quality seemed to be high for this setting and the median follow-up time with more than 7 years seems to be long enough to draw robust conclusions with regard to prognostic effects.

In summary we showed that an SNP in the *RANKL* gene might have an influence on bone metastasis-free survival. This adds to the data that has been published, showing that the RANK/RANKL/OPG pathway plays a crucial role in breast cancer prognosis and bone related events. Further studies will have to examine for which subgroup of patients the findings apply and whether genetic variants have an influence on the efficacy of bone directed therapies.

## Figures and Tables

**Table 1:** Patient characteristics.

Characteristics	Mean or *N*	SD or %
Age	55.7	11.9
BMI	26	4.8
Tumor stage		
pT0	27	2.2
pT1	788	63.0
pT2	360	28.8
pT3	51	4.1
pT4	25	2.0
Nodal status		
pN0	804	64.3
pN+	447	35.7
Tumor type		
Ductal	826	66.0
Lobular	244	19.5
Other	181	14.5
Grading		
G1	130	10.4
G2	835	66.7
G3	286	22.9
ER		
Negative	345	27.6
Positive	906	72.4

**Table 2:** Genotype and allele distribution.

SNP	Chrom.^1^	Position	Alleles^2^	MAF^3^ (%)	Homozygous common^4^	Heterozygous^4^	Homozygous rare^4^
rs1054016 (*RANKL*)	13	43182002	G/T	42.2	427 (34.1)	592 (47.3)	232 (18.5)
rs1805034 (*RANK*)	18	60027241	T/C	47.8	337 (26.9)	632 (50.5)	282 (22.5)
rs35211496 (*RANK*)	18	60021761	C/T	17.3	861 (68.8)	347 (27.7)	43 (3.4)

^1^Chromosome; ^2^major/minor allele, based on the forward strand and minor allele frequency; ^3^minor allele frequency; ^4^frequency, percentage in brackets.

**Table 3:** Bone metastasis-free survival (BMFS), disease-free (DFS), and overall survival (OS) for selected SNPs. Adjusted hazard ratios^1^ (HRs), 95% confidence intervals (CIs), and corresponding *P* values are shown.

SNP	Genotype	BMFS		DFS		OS	
		HR (95% CI)	*P* value	HR (95% CI)	*P* value	HR (95% CI)	*P* value
rs1054016	GG	1 (reference)		1 (reference)		1 (reference)	
	GT	1.06 (0.68, 1.65)	0.79	1.21 (0.88, 1.67)	0.24	1.22 (0.88, 1.69)	0.23
	**TT**	**0.37 (0.17, 0.84)**	**0.02**	0.91 (0.59, 1.40)	0.66	1.00 (0.65, 1.54)	1.00

rs1805034	TT	1 (reference)		1 (reference)		1 (reference)	
	CT	0.73 (0.46, 1.17)	0.19	0.83 (0.60, 1.16)	0.29	0.88 (0.63, 1.24)	0.48
	CC	0.62 (0.34, 1.16)	0.13	0.91 (0.61, 1.35)	0.64	1.09 (0.73, 1.62)	0.67

rs35211496	CC	1 (reference)		1 (reference)		1 (reference)	
	CT	0.90 (0.56, 1.45)	0.67	0.97 (0.70, 1.33)	0.84	1.01 (0.74, 1.39)	0.94
	TT	0.82 (0.20, 3.39)	0.79	1.19 (0.52, 2.73)	0.67	0.73 (0.27, 1.99)	0.54

^1^HRs were adjusted for age, BMI, tumor stage, nodal status, tumor type, grading, and ER.

## References

[1] Coleman R, Gnant M, Paterson A Effects of bisphosphonate treatment on recurrence and cause-specific mortality in women with early breast cancer: a meta-analysis of individual patient data from randomised trials.

[2] Juhasz-Boss I, Fehm T, Ney JT (2012). Pathophysiology of bone remodelling and current therapeutic approaches. *Geburtsh Frauenheilk*.

[3] Asselin-Labat M-L, Vaillant F, Sheridan JM (2010). Control of mammary stem cell function by steroid hormone signalling. *Nature*.

[4] Gonzalez-Suarez E, Jacob AP, Jones J (2010). RANK ligand mediates progestin-induced mammary epithelial proliferation and carcinogenesis. *Nature*.

[5] Jones DH, Nakashima T, Sanchez OH (2006). Regulation of cancer cell migration and bone metastasis by RANKL. *Nature*.

[6] Joshi PA, Jackson HW, Beristain AG (2010). Progesterone induces adult mammary stem cell expansion. *Nature*.

[7] Schramek D, Leibbrandt A, Sigl V (2010). Osteoclast differentiation factor RANKL controls development of progestin-driven mammary cancer. *Nature*.

[8] Fasching PA, Ekici AB, Wachter DL (2013). Breast cancer risk—from genetics to molecular understanding of pathogenesis. *Geburtsh Frauenheilk*.

[9] Heusinger K, Jud SM, Häberle L (2012). Association of mammographic density with hormone receptors in invasive breast cancers: results from a case-only study. *International Journal of Cancer*.

[10] Blair JM, Zhou H, Seibel MJ, Dunstan CR (2006). Mechanisms of disease: roles of OPG, RANKL and RANK in the pathophysiology of skeletal metastasis. *Nature Clinical Practice Oncology*.

[11] Stopeck AT, Lipton A, Body J-J (2010). Denosumab compared with zoledronic acid for the treatment of bone metastases in patients with advanced breast cancer: a randomized, double-blind study. *Journal of Clinical Oncology*.

[12] Coleman RE, Rubens RD (1987). The clinical course of bone metastases from breast cancer. *British Journal of Cancer*.

[13] Colleoni M, O’Neill A, Goldhirsch A (2000). Identifying breast cancer patients at high risk for bone metastases. *Journal of Clinical Oncology*.

[14] Zhang XH-F, Wang Q, Gerald W (2009). Latent bone metastasis in breast cancer tied to Src-dependent survival signals. *Cancer Cell*.

[15] Rosa Mendoza ES, Moreno E, Caguioa PB (2013). Predictors of early distant metastasis in women with breast cancer. *Journal of Cancer Research and Clinical Oncology*.

[16] Smid M, Wang Y, Zhang Y (2008). Subtypes of breast cancer show preferential site of relapse. *Cancer Research*.

[17] Korde LA, Gralow JR (2011). Can we predict who’s at risk for developing bone metastases in breast cancer?. *Journal of Clinical Oncology*.

[18] Lipton A, Chapman J-AW, Demers L (2011). Elevated bone turnover predicts for bone metastasis in postmenopausal breast cancer: results of NCIC CTG MA.14. *Journal of Clinical Oncology*.

[19] Styrkarsdottir U, Halldorsson BV, Gretarsdottir S (2008). Multiple genetic loci for bone mineral density and fractures. *The New England Journal of Medicine*.

[20] Zhang L, Choi HJ, Estrada K (2013). Multistage genome-wide association meta-analyses identified two new loci for bone mineral density. *Human Molecular Genetics*.

[21] Rivadeneira F, Styrkarsdottir U, Estrada K (2009). Twenty bone-mineral-density loci identified by large-scale meta-analysis of genome-wide association studies. *Nature Genetics*.

[22] Estrada K, Styrkarsdottir U, Evangelou E (2012). Genome-wide meta-analysis identifies 56 bone mineral density loci and reveals 14 loci associated with risk of fracture. *Nature Genetics*.

[23] Albagha OME, Wani SE, Visconti MR (2011). Genome-wide association identifies three new susceptibility loci for Paget’s disease of bone. *Nature Genetics*.

[24] Albagha OME, Visconti MR, Alonso N (2010). Genome-wide association study identifies variants at CSF1, OPTN and TNFRSF11A as genetic risk factors for Paget’s disease of bone. *Nature Genetics*.

[25] Teitelbaum SL, Ross FP (2003). Genetic regulation of osteoclast development and function. *Nature Reviews Genetics*.

[26] Fasching PA, Loehberg CR, Strissel PL (2008). Single nucleotide polymorphisms of the aromatase gene (CYP19A1), HER2/neu status, and prognosis in breast cancer patients. *Breast Cancer Research and Treatment*.

[27] Heusinger K, Loehberg CR, Haeberle L (2011). Mammographic density as a risk factor for breast cancer in a German case-control study. *European Journal of Cancer Prevention*.

[28] Rauh C, Hack CC, Haberle L (2012). Percent mammographic density and dense area as risk factors for breast cancer. *Geburtsh Frauenheilk*.

[29] Schrauder M, Frank S, Strissel PL (2008). Single nucleotide polymorphism D1853N of the ATM gene may alter the risk for breast cancer. *Journal of Cancer Research and Clinical Oncology*.

[30] Lambrechts D, Truong T, Justenhoven C 11q13 is a susceptibility locus for hormone receptor positive breast cancer. *Human Mutation*.

[31] Fasching PA, Pharoah PD, Cox A (2012). The role of genetic breast cancer susceptibility variants as prognostic factors. *Human Molecular Genetics*.

[32] Michailidou K, Hall P, Gonzalez-Neira A (2013). Large-scale genotyping identifies 41 new loci associated with breast cancer risk. *Nature Genetics*.

[33] Haiman CA, Chen GK, Vachon CM (2011). A common variant at the TERT-CLPTM1L locus is associated with estrogen receptor-negative breast cancer. *Nature Genetics*.

[34] Ghoussaini M, Fletcher O, Michailidou K (2012). Genome-wide association analysis identifies three new breast cancer susceptibility loci. *Nature Genetics*.

[35] Garcia-Closas M, Couch FJ, Lindstrom S (2013). Genome-wide association studies identify four ER negative-specific breast cancer risk loci. *Nature Genetics*.

[36] Bojesen SE, Pooley KA, Johnatty SE (2013). Multiple independent variants at the TERT locus are associated with telomere length and risks of breast and ovarian cancer. *Nature Genetics*.

[37] Antoniou AC, Wang X, Fredericksen ZS (2010). A locus on 19p13 modifies risk of breast cancer in BRCA1 mutation carriers and is associated with hormone receptor-negative breast cancer in the general population. *Nature Genetics*.

[38] Vachon CM, Scott CG, Fasching PA (2012). Common breast cancer susceptibility variants in LSP1 and RAD51L1 are associated with mammographic density measures that predict breast cancer risk. *Cancer Epidemiology, Biomarkers & Prevention*.

[39] Grambsch PM, Therneau TM (1994). Proportional hazards tests and diagnostics based on weighted residuals. *Biometrika*.

[40] Pfitzner BM, Branstetter D, Mergler B RANK expression is prognostic and predictive in primary breast carcinoma: analysis of samples from the GeparTrio study.

[41] Santini D, Schiavon G, Vincenzi B (2011). Receptor activator of NF-kB (rank) expression in primary tumors associates with bone metastasis occurrence in breast cancer patients. *PLoS ONE*.

[42] Owen S, Ye L, Sanders AJ (2013). Expression profile of receptor activator of nuclear-kappaB [RANK], RANK ligand [RANKL] and osteoprotegerin [OPG] in breast cancer. *Anticancer Research*.

[43] Ney JT, Fehm T, Juhasz-Boess I (2012). RANK, RANKL and OPG expression in breast cancer—influence on osseous metastasis. *Geburtsh Frauenheilk*.

[44] Lu Y, Liu P, Recker RR, Deng H-W, Dvornyk V (2010). TNFRSF11A and TNFSF11 are associated with age at menarche and natural menopause in white women. *Menopause*.

[45] Park JW, Ji YI, Choi Y-H (2009). Candidate gene polymorphisms for diabetes mellitus, cardiovascular disease and cancer are associated with longevity in Koreans. *Experimental and Molecular Medicine*.

[46] Dong S-S, Liu X-G, Chen Y (2009). Association analyses of RANKL/RANK/OPG gene polymorphisms with femoral neck compression strength index variation in caucasians. *Calcified Tissue International*.

[47] Assmann G, Koenig J, Pfreundschuh M (2010). Genetic variations in genes encoding RANK, RANKL, and OPG in rheumatoid arthritis: a case-control study. *Journal of Rheumatology*.

[48] Ney JT, Juhasz-Boess I, Gruenhage F (2013). Genetic polymorphism of the OPG gene associated with breast cancer. *BMC Cancer*.

[49] Barrett LW, Fletcher S, Wilton SD (2012). Regulation of eukaryotic gene expression by the untranslated gene regions and other non-coding elements. *Cellular and Molecular Life Sciences*.

[50] Fasching PA, Gayther S, Pearce L (2009). Role of genetic polymorphisms and ovarian cancer susceptibility. *Molecular Oncology*.

[51] Pharoah P (2006). Commonly studied single-nucleotide polymorphisms and breast cancer: results from the Breast Cancer Association Consortium. *Journal of the National Cancer Institute*.

